# Correlation between macular vessel density and number of intravitreal anti-VEGF agents for macular edema associated with branch retinal vein occlusion

**DOI:** 10.1038/s41598-019-52732-2

**Published:** 2019-11-08

**Authors:** Ryo Tomita, Takeshi Iwase, Kensuke Goto, Kentaro Yamamoto, Eimei Ra, Hiroko Terasaki

**Affiliations:** 1 Department of Ophthalmology, Nagoya University Graduate School of Medicine, Nagoya, Japan; 2 Department of Ophthalmology, Akita University Graduate School of Medicine, Akita, Japan

**Keywords:** Medical imaging, Outcomes research

## Abstract

We evaluated whether the reduction of macular vessel density was correlated with the number of intravitreal injections of anti-vascular endothelial growth factor (VEGF) agents in eyes with a branch retinal vein occlusion (BRVO). The mean vessel density was determined by optical coherence tomography angiography in 29 eyes with macular edema associated with a BRVO. Our results showed that the mean vessel density in the group that had a resolution of the macular edema after one anti-VEGF injection was significantly higher than group that had a recurrence of the macular edema (*P* = 0.028). Single regression analysis showed that the number of intravitreal injections was significantly correlated with the reduction of the modified vessel density (*r* = −0.421, *P* = 0.023) and systemic hypertension (*r* = 0.377, *P* = 0.044). Multiple stepwise regression analysis showed that the reduction of the modified vessel density (*β* = −0.442, *P* = 0.009) and hypertension (*β* = 0.403, *P* = 0.016) were independent factors associated with the number of intravitreal injections. We conclude that the vessel density reduction can be used to predict whether recurrences of the macular edema will develop after the initial anti-VEGF injection in eyes with macular edema associated with a BRVO.

## Introduction

A branch retinal vein occlusion (BRVO) is a relatively common retinal vascular disorder in elderly individuals, and it can lead to visual dysfunction^1^. The most frequent cause of the visual impairments in eyes with a BRVO is macular edema^2^. There are several treatment options for the macular edema, e.g., laser photocoagulation^3,4^, intravitreal injection of steroids^5^, subtenon injection of triamcinolone acetonide (STTA)^6^, dexamethasone intravitreal implant^7^, and vitrectomy^8^. It was recently reported that the intravitreal injections of anti-vascular endothelial growth factor (VEGF) agents, such as bevacizumab^9,10^, ranibizumab^11^, or aflibercept^12^, were more effective in resolving the macular edema and improving the best-corrected visual acuity (BCVA) than the other treatments.

However, frequent recurrences of the macular edema can occur after an anti-VEGF injection and repeated injections are required^13^. Accordingly, clinicians want to know how many intravitreal injections will be necessary to achieve a complete remission of the macular edema at the early phase of the treatment regimen.

Several factors have been reported to be associated with the recurrences of the macular edema after the injection of an anti-VEGF agent, e.g., initial central retinal thickness (CRT), age, and several other factors^14–19^. However, it is still difficult to predict the number of recurrences even with knowledge of these factors.

The recent development of optical coherence tomography angiography (OCTA) has allowed clinicians to measure the retinal and choroidal blood flow noninvasively. Importantly, earlier studies have shown that OCTA is a reliable technique to evaluate vascular disorders including those associated with a BRVO^20–22^.

It has been reported that eyes with incomplete vessel loss were more likely to have recurrences of the macular edema than eyes with severe vessel loss in eyes with BRVO^17,18,23,24^. In addition, eyes with a greater reduction in the vessel density determined by OCTA have fewer recurrences of the macula edema^16,18^. However, the exact relationship between the vessel loss and the recurrences of macular edema in eyes with a BRVO has not been definitively determined.

Thus, the purpose of this study was to determine the vessel density and the rate of recurrences of the macular edema after an initial anti-VEGF injection in eyes with macular edema associated with a BRVO. To accomplish this, we used OCTA to determine the vessel density in eyes before and after an anti-VEGF injection. A recurrence of the macular edema was considered to have occurred when the central retinal thickness was > 300 µm.

## Results

### Demographics of patients

Three eyes were excluded due to a prior vitrectomy; one eye that had a BRVO in both the superior and inferior hemispheres and 1 eye that had diabetic retinopathy. In the end, 29 eyes with macular edema secondary to a BRVO and treated with intravitreal injections of anti-VEGF agents were studied. The baseline clinical characteristics of the 29 eyes are summarized in Table 1. The mean age was 66.7 ± 11.5 years with a range of 46–87 years. The mean pre-treatment BCVA was 0.30 ± 0.27 logMAR units (20/40) and the mean pre-treatment CFT was 445.1 ± 112.0 µm. Six eyes were treated with intravitreal ranibizumab (IVR), and 23 were treated with intravitreal aflibercept (IVA).

**Table 1:** Baseline characteristics of subjects.

Characteristic	Mean ± SD
*n* (eyes)	29
Age (years)	66.7 ± 11.5
Sex (male/female)	15/14
IOP (mmHg)	14.1 ± 4.1
Axial length (mm)	23.7 ± 1.2
Visual acuity (LogMAR)	0.30 ± 0.27
CFT (μm)	445.1 ± 112.0
Systolic blood pressure (mmHg)	149.6 ± 24.4
Diastolic blood pressure (mmHg)	88.6 ± 12.9
Hypertension (patients)	11
Dyslipidemia (patients)	3
Diabetes mellitus (patients)	1

IOP: intraocular pressure, LogMAR: logarithm of the minimum angle of resolution, CFT: central foveal thickness.

### Total number of intravitreal injections

The mean of the total number of the intravitreal injection was 1.9 ± 1.1 times in the 29 eyes. The resolved group had 13 eyes, and the recurrence group had 16 eyes. Eleven eyes had two, 3 eyes had three, 1 eye had four, and 1 eye had 6 repeated intravitreal injection of IVR or IVA. The mean of the total number of the injections in the recurrence group was 2.6 ± 1.1.

### Comparisons between resolved group and recurrence group

The clinical characteristics of the resolved group and the recurrence group are summarized in Table 2. The mean interval from the diagnosis of macular edema to the first intravitreal injection was 8.1 ± 8.1 weeks in the resolved group and 6.1 ± 8.0 weeks in the recurrence group. A small amount of subretinal macular hemorrhage was observed in one case in both groups. There were no significant differences in the clinical characteristics except that the mean age of the recurrence group was significantly older than the resolved group (*P = *0.021).

**Table 2:** Baseline characteristics of resolved group and recurrence group.

Parameter	Resolved group	Recurrence group	*P* Value
*n* (eyes)	13	16	-
**Baseline**			
Age (years)	61.3 ± 10.7	71.2 ± 10.9	0.021
Sex (male/female)	5/8	10/6	0.198
Hypertension (patients)	3	8	0.135
Dyslipidemia (patients)	0	3	0.153
Diabetes mellitus (patients)	0	1	0.552
Period from diagnosis to first injection (weeks)	8.12 ± 8.13	6.14 ± 8.03	0.517
Visual acuity (LogMAR)	0.35 ± 0.37	0.26 ± 0.17	0.428
CFT (μm)	455 ± 127	438 ± 106	0.696
**One month after first injection**			
Visual acuity (LogMAR)	0.24 ± 0.34	0.15 ± 0.14	0.362
CFT (μm)	215 ± 37.2	230 ± 27.9	0.223
Vessel density reduction rate (%)	19.8 ± 28.1	5.87 ± 17.2	0.113
Modified vessel density reduction rate (%)	37.4 ± 27.5	17.5 ± 18.4	0.028
Area of FAZ (mm^2^)	0.32 ± 0.09	0.25 ± 0.10	0.070
**Six month after first injection**			
Visual acuity (LogMAR)	0.18 ± 0.34	0.11 ± 0.12	0.524
Total number of injections (times)	1.0	2.56 ± 1.09	< 0.001

LogMAR: logarithm of the minimum angle of resolution, CFT: central foveal thickness, FAZ: foveal avascular zone.

### Values of different parameters obtained by OCTA

**Figure 1:**
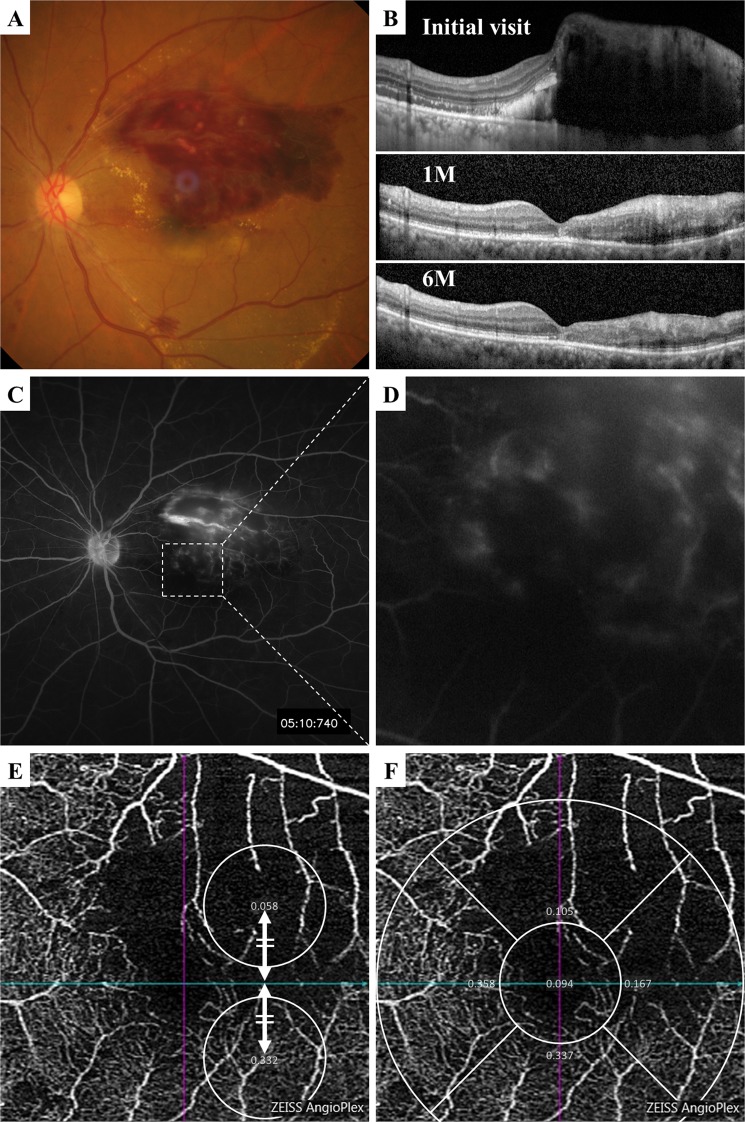
Representative case with macular edema secondary to a branch retinal vein occlusion (BRVO) that was resolved by single intravitreal injection of anti-VEGF agent during the follow-up period. (**A**) Fundus photograph showing major BRVO at the first visit. (**B**) Vertical OCT image shows the macular edema at the initial visit, but the edema was resolved throughout the follow-up period after the anti-VEGF injection. (**C,D**) Fluorescein angiogram showing sparse hyperfluorescence corresponding to the occluded region at the initial visit. (**E**) OCTA image showing wide non-perfused area in the occluded area, and the vessel density was significantly lower in the affected area at 1 month after the first injection. The modified vessel density reduction was 82.5%. (**F**) The vessel density reduction rate was 68.8%.

**Figure 2:**
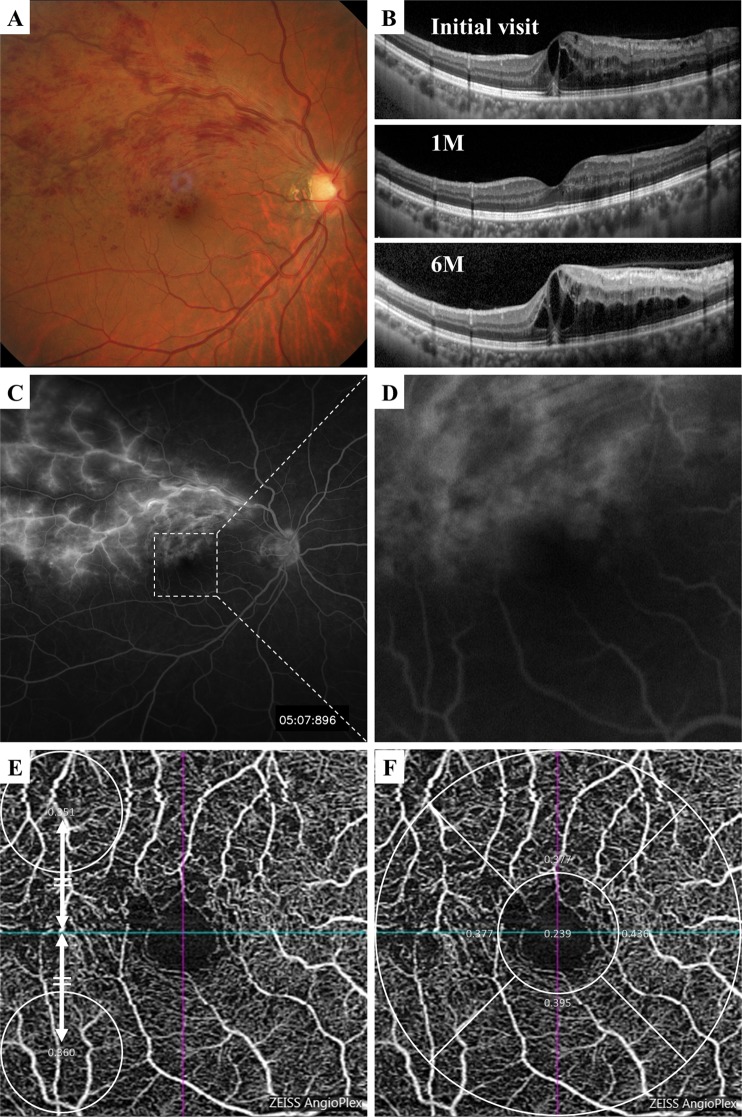
Representative case of macular edema secondary to a BRVO that required four intravitreal injections of anti-VEGF agent during the 6 months follow-up period. (**A**) Fundus photograph showing major BRVO at first visit. (**B**) Vertical OCT image showing macular edema at the initial visit, and the edema was resolved at 1 month after the first injection but recurred. (**C,D**) Fluorescein angiogram showing massive hyperfluorescence at the initial visit. (**E**) OCTA image showing macular region at 1 month after the first injection. The modified vessel density reduction rate was 2.5% (**E**), and the vessel density reduction rate was 4.6% (**F**).

**Figure 3:**
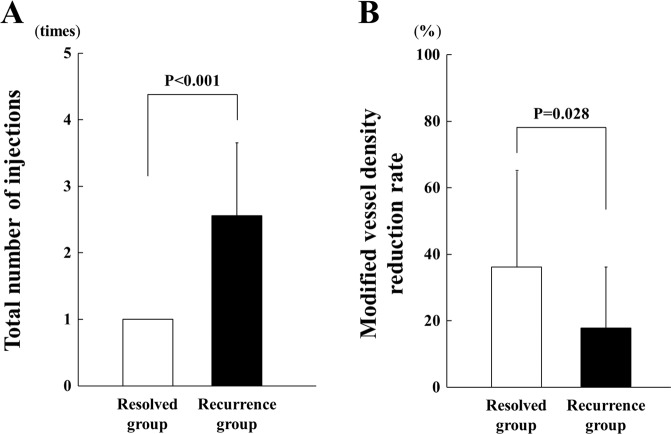
Total number of anti-VEGF injections during the follow-up period was 1.0 in the resolved group and 2.56 ± 1.09 in the recurrence group, and it was significantly different between the groups (P < 0.001). (**A**) The modified vessel density reduction rate was 37.4 ± 27.5% in the resolved group and 17.5 ± 18.4% in the recurrence group, and the difference was significant (*P* = 0.028) (**B**).

The mean superficial FAZ area after the first injection was 0.28 ± 0.10 mm^2^. The mean reduction in the vessel density was 19.8 ± 28.1% in the resolved group (Fig. 1) and 5.87 ± 17.2% in the recurrence group (Fig. 2) at 1 month after the first intravitreal injection. The difference in the mean percentage reduction between the two groups was not significant. On the other hand, the mean modified vessel density reduction was 37.4 ± 27.5% in the resolved group which was significantly higher the 17.5 ± 18.4% in the recurrence group (*P = *0.028; Fig. 3).

### Correlations between number of injections and other parameters

**Figure 4:**
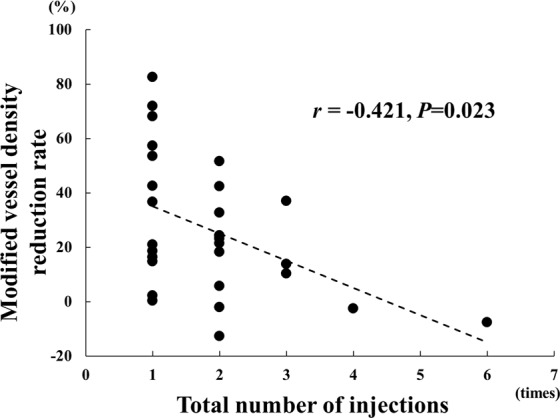
Relationship between the total number of intravitreal injections of anti-VEGF agents until 6 months after the first injection and the modified vessel density reduction rate 1 month after first injection. The total number of intravitreal injections was significantly correlated with the modified vessel density reduction rate (r = −0.421, *P* < 0.023).

**Figure 5:**
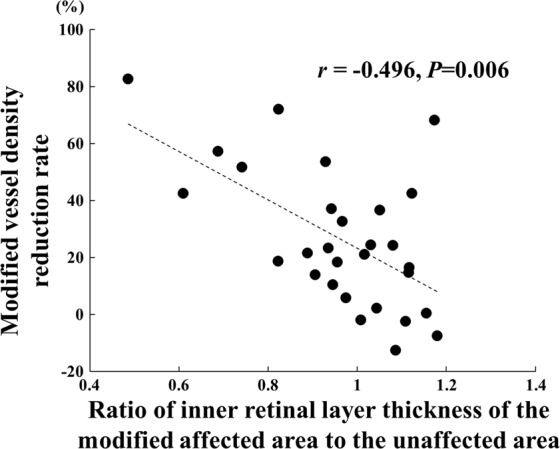
The reduction of the modified vessel density was significantly correlated with the ratio of inner retinal layer thickness of the modified affected area to the unaffected area where the modified vessel density rate was measured using OCTA. (*r* = −0.496, *P* = 0.006).

Single regression analyses showed that the number of the intravitreal injections was significantly correlated with the reduction of the modified vessel density (*r* = -0.421, *P* = 0.023) and with the systemic hypertension (*r* = 0.377, *P* = 0.044; Table 3; Fig. 4). However, the reduction in the vessel density, the pre-treatment BCVA, and the pre-treatment CFT were not significantly correlated with the number of injections. The reduction of the modified vessel density was significantly correlated with the ratio of inner retinal layer thickness of the modified affected area to the unaffected area (*r* = -0.496, *P* = 0.006; Fig. 5). Multiple stepwise regression analyses showed that the reduction in the modified vessel density (*β* = -0.442, *P* = 0.009) and systemic hypertension (*β* = 0.403, *P* = 0.016) were independent factors significantly associated with the number of injections (Table 4).

**Table 3:** Results of spearman’s rank correlation coefficient between the total number of injections and clinical parameters.

Parameters		*r*	*P* Value
Total number of injections	Modified vessel density reduction rate	−0.421	0.023
	Hypertension	0.377	0.044
	Sex	−0.357	0.057
	Dyslipidemia	0.307	0.105
	CFT 1 month after first injection	0.284	0.136
	Vessel density reduction rate	−0.275	0.149
	Age	0.231	0.227
	FAZ	−0.222	0.247
	Systolic blood pressure	−0.172	0.410
	Baseline visual acuity	−0.134	0.487
	Diabetes mellitus	0.098	0.614
	Baseline CFT	−0.042	0.828
	Visual acuity 1 month after first injection	−0.033	0.864

CFT: central foveal thickness, FAZ: foveal avascular zone.

**Table 4:** Results of multiple stepwise regression analysis for independence of factors contributing to the number of injections.

Variable		*β*	*P* Value
Dependent	Independent		
Total number of injections	Modified vessel density reduction rate	−0.442	0.009
	Hypertension	0.403	0.016
	Sex	−0.225	0.180
	CFT 1 month after first injection	0.169	0.315
	Dyslipidemia	0.087	0.601
	Diabetes mellitus	−0.086	0.601
	FAZ	−0.063	0.706
	Visual acuity 1 month after first injection	0.026	0.879
	Age	0.009	0.957

CFT: central foveal thickness, FAZ: foveal avascular zone.

## Discussion

We evaluated the reduction of macular vessel density and found that the reduction of the modified vessel density in the resolved group was significantly higher than that in the recurrence group. It was also significantly correlated with the number of intravitreal anti-VEGF injections during the follow-up period. These findings indicate that the eyes with a greater loss of macular capillaries were more likely to have a fewer number of recurrences of macular edema and fewer number of injections.

The current strategy for the treatment of macular edema secondary to a BRVO is mainly intravitreal injection of anti-VEGF agents. Actually, many large-scale clinical trials have clearly demonstrated a rapid resolution of macular edema and a significant improvement of the visual acuity by the anti-VEGF agents compared with other treatment, e.g. STTA or laser treatment^25–27^. However, one of the inherent problems of anti-VEGF therapy is that frequent recurrences can occur within a period of a few months after the injection and multiple injections of the agents are required for the recurrences in many cases^13,26,28^. Accordingly, it is important to determine a method to predict recurrences at the early phase of the treatment. This would then allow the clinician to strategize the best treatment protocol.

Several factors have been reported to be related to the recurrences of macular edema in eyes with BRVO, e.g., the CRT^15,19^. However, the CRT was not significantly correlated with the number of injection and no significant difference in the CRT was found between the resolved and recurrence group. It has been reported that the vessel density was decreased in both the superficial and deep vessel capillary plexuses^29^, and the gap between the surface and deep capillary plexus is significantly greater in the recurrent macular edema group^16^. In addition, Hasegawa *et al*. divided the 3 × 3 mm image obtained by OCTA at 1 month after IVR into superior and inferior sectors and calculated the percentage of reduction in the vessel density by dividing the affected sector by the unaffected sector. They found that eyes with a larger degree of vessel reduction had fewer recurrences and lower percentage of IVR injections for 12 months in eyes with BRVO^18^. However, the affected sector included areas with normal vasculature and normal FAZ as well as the unaffected sector. We evaluated the vessel density reduction percentage by comparing the affected sector (1.57 mm^2^) and not affected sector using the ETDRS grid, which is smaller than the area (4.5 mm^2^) reported by Hasegawa *et al*. However, our results showed no significant difference in the vessel density reduction rate between the resolved group and the recurrence group, and no significant correlation between the vessel density reduction rate and the number of injections. One of the reasons for the lack of a significant difference between the groups is that the area still included areas with normal vasculature even in the affected sector, although the measured area was smaller than that reported by Hasegawa^17,18^. This situation could lead to an underestimation in the degree of vessel density reduction and result in no significant difference in the vessel density reduction between the two groups. These findings indicate that it is not easy to evaluate accurately the vessel reduction in the macular region in eyes with a BRVO.

Then, the center of the ETDRS grid (0.79 mm^2^) was placed at the region where the retinal vessel density of the affected retina was the lower, and we determined the modified vessel density reduction rate. Our results showed that the degree of reduction of the modified vessel density in the resolved group was higher than that in the recurrence group, and it was significantly correlated with the number of anti-VEGF injections.

It is more likely that the modified vessel density reduction is related to the degree of vessel disruption because the measured area was smaller and included only the occluded region in most eyes. Actually, previous studies have reported that a severe reduction in the macular vasculature may cause the elimination of the source of the leakage which would then be fewer recurrence of macular edema^24^. In addition, eyes with a greater defect of macular capillaries may lead to atrophy of the inner retinal layer, lower oxygen demand, and lower VEGF production^18,30,31^. In fact, our study showed that the modified vessel density rate was significantly correlated with the ratio of inner retinal layer thickness on the affected area to the unaffected area 6 months after the first injection. This result suggests that eyes with a larger vessel density reduction should have fewer leaking vessels which is the cause of the macular edema and may reduce oxygen consumption in the inner retina leading to a lower rate of recurrences and fewer anti-VEGF injections. Taken together, the modified vessel density reduction at 1 month after initial the anti-VEGF injection would be a useful parameter to predict the number of anti-VEGF injections at the early phase of the treatment in eyes with BRVO.

Our result showed that a history of systemic hypertension was correlated with the number of anti-VEGF injections. It has been reported that hypertension is a risk factor for the development of a RVO^32,33^. Kida reported three cases in which there was an improvement of the macular edema after a treatment of only the systemic hypertension in patients with macular edema secondary to RVO^34^. These results suggest that RVO might recur more often in patients with hypertension. Our patients with hypertension were treated, and the blood pressure was decreased to normal range during the course of this study. This might explain the observation that the blood pressure was not significantly correlated with the number of injections.

Our study has several limitations. First, this was a retrospective study. Second, intravitreal injections were not unified with the use of either aflibercept or ranibizumab. Third, we selected the location to measure the modified blood vessel density reduction rate manually. Fourth, we did not evaluate the leakage from the vessels using FA, and thus the association between the time of injection and the leakage is unclear. Fifth, the evaluations of the retinal vasculature in OCTA is performed only on the superficial capillary plexus. However, the resolution in deep layer detected by commercially available OCTA is still not good, and it is difficult to obtain accurate evaluations using the deep capillary plexus parameters. Further prospective studies using a larger number of subjects using one anti-VEGF agent and the analyses of both the superficial and deep capillary areas determined by OCTA will be necessary to confirm the association between the retinal vasculature and the number of injection of anti-VEGF agents.

In conclusion, the modified vessel reduction rate was higher in eyes that had a resolution of the macular edema after a single injection of an anti-VEGF agent. It was significantly negatively correlated with the total number of intravitreal injections. These results indicate that the modified vessel density rate would be a useful parameter to use to predict the number of anti-VEGF injections for macular edema secondary to a BRVO.

## Patients and Methods

### Ethics statement

This was a single-center, retrospective study, and the procedures were approved by the Ethics Committee of the Nagoya University Hospital, Nagoya, Japan. The procedures conformed to the tenets of the Declaration of Helsinki. A signed written informed consent was obtained from all patients for the intravitreal injections.

### Patients

All of the patients were examined at the Nagoya University Hospital between January 2016 and May 2018. The medical records of patients with macular edema that was associated with a BRVO, and whose spectral-domain OCT (SD-OCT) images showed that the edema extended to the fovea were analyzed. The macular edema was treated with intravitreal ranibizumab (IVR) or aflibercept (IVA) injections. The patients underwent the OCTA examinations when the macular edema was resolved at 1 month after the first intravitreal injection. They were then followed for at least 6 months after the injection, and they received repeat IVR or IVA when the central foveal thickness was ≥300 μm. The same anti-VEGF agent as the first injection was injected to treat the recurrence of the macular edema.

The patients were classified into 2 groups; patients who had only one intravitreal injection were classified as the resolved group, and patients who required more than one intravitreal injection were classified as the recurrence group. The total number of intravitreal injections after the first injection for 6 months was used for the statistical analyses.

All of the patients had a comprehensive ophthalmic examination including measurement of the best-corrected visual acuity (BCVA), fundus examinations by indirect ophthalmoscopy, slit-lamp biomicroscopy, and fundus photography. The Snellen BCVA values were converted to the logarithm of the minimum angle of resolution (logMAR) units for the statistical analyses.

### Exclusion criteria

Patients with other macular diseases, such as age-related macular degeneration, myopic choroidal neovascularization, epiretinal membrane, diabetic retinopathy, history of other ocular diseases, rhegmatogenous retinal detachment, and severe cataract, were excluded.

### Retinal microvasculature imaging by OCTA

The Angio Plex^®^OCTA (Zeiss Meditec. Inc, Germany) was used to obtain 3 × 3-mm en face images of the microvasculature centered on the fovea. The boundaries of the superficial capillary plexus extended from 3 µm beneath the internal limiting membrane (ILM) to 15 µm beneath the inner plexiform layer (IPL). All of the scans were reviewed by two graders (RT and TI), and the eyes with poor image quality caused by blink artifacts, poor fixation leading to motion or doubling artifacts, and media opacities that obscured the view of the vasculature, were excluded.

The superficial OCTA image of 3 mm diameter centered on the fovea was automatically divided into 5 sectors, e.g., the fovea of 0.79 mm^2^, the inferior, superior, nasal, and temporal (all, 1.57 mm^2^) sectors as defined by the Early Treatment Diabetic Retinopathy Study (ETDRS) grid. The vessel density in each of the ETDRS sector was automatically measured by the embedded software of the OCTA device (version 10).

The ETDRS grid was centered on the fovea, and the vessel density of the superior or inferior sectors that was affected by the BRVO was defined as the affected vessel density sectors and the vessel density of the unaffected sectors was defined as the unaffected vessel density^17^.

The reduction in the vessel density (%) was calculated as:

$$Reduction\,of\,vessel\,density\,( \% )=(1-affected\,vessel\,density/unaffected\,vessel\,density)\times 100.$$

We then displaced the center of the ETDRS grid to the area where the vessel density was most decreased on the affected hemisphere and defined the vessel density of the area as the modified affected vessel density. In addition, we measured it at a horizontally symmetrical position containing fovea and defined this as the modified unaffected vessel density. The reduction in the modified vessel density (%) was defined as;

$$Reduction\,of\,modified\,vessel\,density\,( \% )=(1-modified\,affected\,vessel\,density/modified\,unaffected\,vessel\,density)\times 100.$$

The areas of the superficial foveal avascular zone (FAZ) were determined after the resolution of the macular edema with the software embedded in the OCTA device.

### Analyses of OCT images

A Spectralis^®^ SD-OCT instrument (Heidelberg Engineering, Heidelberg, Germany) was used to obtain horizontal cross-sectional images that were recorded before and 1 month after the treatment. The central foveal thickness (CFT) was defined as the thickness between the surface of the ILM and the outer border of the retinal pigment epithelium (RPE) centered on the fovea. Six months after the first injection, the ratio of the inner retinal layer thickness at the center of the affected area to the unaffected areas where the modified vessel density rate was measured using OCTA.

### Statistical analyses

The values are presented as the means ± standard deviations (SDs). Spearman’s rank tests were used to determine the significance of the correlation coefficients between each parameter and the number of intravitreal injections. Forward stepwise regression analyses were performed to identify the factors significantly correlated with the number of the intravitreal injections. Independent sample *t* tests, nonparametric Mann-Whitney U tests, and Chi-squared tests were used to determine the significance of the differences in the variables between the resolved group and the recurrence group. All statistical analyses were performed with the Statistical Package for Social Sciences for Windows 25.0 (SPSS Inc, Chicago, Illinois, USA).
